# Predicting the Efficacy of HER2-Targeted Therapies: A Look at the Host

**DOI:** 10.1155/2017/7849108

**Published:** 2017-12-18

**Authors:** Martina Di Modica, Elda Tagliabue, Tiziana Triulzi

**Affiliations:** Molecular Targeting Unit, Department of Research, Fondazione IRCCS Istituto Nazionale dei Tumori, Milan, Italy

## Abstract

HER2 is overexpressed in 20% of invasive breast cancers (BCs) and correlates with a more aggressive disease. Until the advent of targeted agents, HER2 was associated with worse outcomes. Rationally designed HER2-targeted agents have been developed and introduced into clinical practice for women with HER2-amplified BC, improving disease-free and overall survival for primary and metastatic tumors. Trastuzumab, a recombinant humanized anti-HER2 monoclonal antibody, combined with chemotherapy, remains the standard of care for patients with HER2-positive BCs. However, many patients do not respond to this agent, whereas newer drugs have proven to be efficacious in clinical trials. The identification of biomarkers that select sensitive tumors and patients who will benefit from these new agents would help the incorporation of these therapies, limiting the risk of side effects and overtreatment and improving the outcomes of all patients with early-stage HER2-positive BC. We review the mechanisms of action of HER2-targeting agents, focusing on the involvement of the immune system and related predictive biomarkers.

## 1. Introduction

The tyrosine kinase HER2, with the other members of the HER (human EGF receptor) family of receptor tyrosine kinases (i.e., HER1, HER3, and HER4), controls many signaling pathways in various cellular functions, including proliferation, migration, survival, DNA repair, and angiogenesis (reviewed in [1]). Based on its oncogenic function, tumors in which this oncogene is amplified, constituting 20% of breast cancers (BCs), treated with conventional chemotherapy alone are aggressive, lead to early relapse, and have a bad prognosis [2].

Over the past 15 years, significant progress has been made in the clinical management of BC patients, with the introduction of rationally designed targeted agents [3]. Today, patients with HER2-positive BC who are treated with trastuzumab typically experience better outcomes than those with HER2-negative disease [4]. Several strategies have been adopted to target the HER2 oncogene: monoclonal antibodies (MAbs) that bind to the extracellular domain of HER2 (such as trastuzumab and pertuzumab); antibody-drug conjugates (such as trastuzumab emtansine, called T-DM1); and tyrosine kinase inhibitors (TKIs) (such as lapatinib and neratinib), which compete with the ATP-binding site of the catalytic domain of HERs.

The recombinant humanized MAb trastuzumab binds to the juxtamembrane region (subdomain IV) of HER2 and, with or without chemotherapy, is the basis for systemic treatment of metastatic and early HER2-positive BC [5]. Although trastuzumab remains the standard treatment, other anti-HER2 agents have been approved for advance disease and tested for early disease in several trials: pertuzumab, a fully humanized recombinant MAb that targets the extracellular dimerization domain (subdomain II) of HER2, blocking its dimerization with HER1 and HER3; TDM1, comprising trastuzumab that is linked to the cytotoxic agent emtansine (DM1); lapatinib, a reversible TKI of HER2 and EGFR that competes with ATP for the ATP-binding pocket; and neratinib, a pan-HER inhibitor that interacts covalently with a conserved cysteine residue in HERs.

Despite the therapeutic options that are available, the clinical benefit of trastuzumab and its combination with other HER2-targeted therapies that have complementary mechanisms of action—the dual blockade approach—remain modest, with many patients who do not improve survival with these agents. Many studies have been performed on the mechanisms of trastuzumab action and the efficacy and resistance of second-generation HER2-targeting compounds to identify patients in whom the therapeutic effects of these tailored therapies can be optimized.

We review mechanisms of action of these drugs, focusing on the involvement of the immune system and the related biomarkers that have potential value in selecting patients for the most appropriate treatment option in neoadjuvant-adjuvant settings.

## 2. Mechanisms of Action of HER2-Targeting Agents

**Figure 1:**
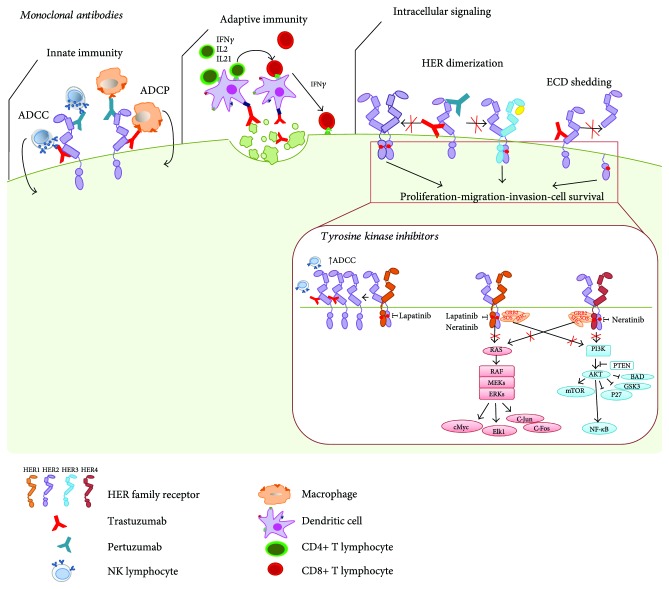
Anti-HER2 therapies and their immunostimulatory properties. The monoclonal antibodies trastuzumab and pertuzumab, in addition to inhibiting intracellular signaling downstream of HER2 activation (i.e., homo/heterodimerization and proteolytic cleavage of the HER2 extracellular domain), induce an antitumor immune response in the tumor microenvironment. Trastuzumab and pertuzumab bind to the extracellular domain of HER2 and, through their Fc portions, engage antibody-dependent cell-mediated cytotoxicity (ADCC) and phagocytosis (ADCP) in Fc receptor-positive innate immune cells (i.e., NK lymphocytes, macrophages, monocytes, and neutrophils). Immune complex and opsonized tumor fragments are recognized and taken up by dendritic cells via the Fc receptor. Dendritic cells and other antigen-presenting cells (e.g., macrophages) present tumor antigens through MHC-II molecules to CD4+ T-helper lymphocytes, which release interferon-*γ* (IFN*γ*), interleukin 2 (IL2), and IL21 to enhance the cytotoxic T cell response. Antigens presented by MHC-I molecules directly stimulate CD8+ cytotoxic T lymphocytes. CD8+ T cells can also recognize tumor antigens presented on MHC-I molecules by cancer cells themselves and initiate a cytotoxic response. Tyrosine kinase inhibitors (TKIs) (lapatinib and neratinib) block the kinase domain activity of HERs, disrupting the oncogenic signals that lead to proliferation, migration, invasion, and survival of cancer cells. In contrast to neratinib, lapatinib, in addition to blocking the TK domain of HER2 and HER1, affects the accumulation of HER2 on the surface of BC cells, leading to an increase in ADCC when combined with trastuzumab.

The treatment of HER2-positive BC with MAbs and TKIs aims to impede uncontrolled tumor growth and invasiveness by blocking the intracellular signals that are derived from HER2. In addition to inhibiting oncogenic stimuli, the efficacy of HER2-targeting agents in BC is also based on their ability to engage antitumor immunity (Figure 1).

### 2.1. Monoclonal antibodies

Trastuzumab (Herceptin, Genentech, South San Francisco, CA, USA) uses several mechanisms to block HER2 signaling [6] (Figure 1), underlying the cytostatic activity of this anti-HER2 agent [7]. It blocks the intracellular mitogen-activated protein kinase cascade (RAS/RAF/MEK/ERK) and the phosphoinositide 3-kinase (PI3K/AKT/mTOR) pathway, arresting the cell cycle in G1 phase [8], and promotes ubiquitination, endocytosis, and degradation of HER2, decreasing its expression on the tumor cell surface [9, 10].

Moreover, trastuzumab inhibits the proteolytic cleavage of HER2, preventing shedding of the extracellular domain (ECD) and the generation of phosphorylated truncated p95-HER2, which has been implicated in tumor growth and progression [11]; this activity is reflected by the significant decrease of HER2 ECD in serum of patients in clinical trials after treatment [12, 13]. Based on the involvement of HER2 signaling in controlling the expression of pro- and antiangiogenic factors, trastuzumab decreases the volumes of blood vessels in SCID mice that bear HER2-positive BC tumors [14].

One of the most significant mechanisms of action of trastuzumab is the antibody-dependent cellular-mediated cytotoxicity (ADCC) (Figure 1). Trastuzumab-coated cells are recognized through its Fc region by immune cells that express Fc receptor (Fc*γ*R) (e.g., NK cells, macrophages, neutrophils, and eosinophils). The efficacy of trastuzumab is lost in Fc*γ*R-deficient mice and on inhibition of Fc*γ*R engagement in preclinical models [15]. Similarly, a loss-of-function polymorphism in Fc*γ*R reduces the efficacy of trastuzumab in BC patients [16, 17]. NK cells constitutively express Fc*γ*RIIIA (CD16) and are the major effectors of ADCC. Consistently, it has been reported that patient response to trastuzumab monotherapy is associated with robust tumor infiltration of lymphoid cells [18]—primarily NK cells [19]—and a greater capacity of NK cells to mediate *in vitro* antibody-dependent cellular cytotoxicity [20, 21]. We recently demonstrated the involvement of NKG2D in the induction of trastuzumab-mediated ADCC by NK cells [21], which could explain the synergism between trastuzumab and taxanes in clinical trials [22]—NKG2D expression on NK cells is increased by taxanes and is associated with NK cytotoxic activity [21]. Moreover, NKG2D receptor on NK cells binds to MICA, one of its ligands, on monocytes that reside in the tumor microenvironment, boosting NK cell antitumor activity against Ab-coated tumor cells and ultimately increasing their production of interferon-*γ* (IFN*γ*) [23].

Another recent study reported that trastuzumab induces antibody-dependent cellular phagocytosis- (ADCP-) mediated cell death through recognition of trastuzumab-opsonized cells by Fc*γ*R on macrophages (Figure 1). Anti-HER2 increased the percentage of systemic and tumor-infiltrating macrophages in BT474 xenografts, and their depletion prior to trastuzumab treatment by clodrosome significantly impaired its ability to inhibit tumor growth [24]. Similarly, depletion of CD11b+ cells in BALB/c mice that bear H2N100 tumors limits the activity of anti-HER2 therapy [25], also implicating macrophages in the antitumor efficacy of trastuzumab.

Other studies that have aimed to determine the function of the immune system in trastuzumab activity revealed a critical role of adaptive immune cells [25–27]. Anti-HER2 MAbs have a limited impact on tumor growth in Rag1^−/−^ mice, which lack T and B lymphocytes [26], and the depletion of CD8+ T cells abrogates its antitumor activity in BALB/c mice bearing TUBO or H2N100 tumors [25, 26]. CD8+ T cells mediate the protection against tumor rechallenge, based on their ability to establish a long-term immune memory in mice that are treated with anti-HER2 MAbs [25, 26]. Accordingly, CD8+ T cell numbers rise in patients after trastuzumab treatment [26].

Stagg et al. demonstrated that trastuzumab depends in part on the antitumor effects of IFNs using neutralizing antibodies to IFN*γ* and IFNAR1 (type I IFN receptor) [25]. They proposed a model in which trastuzumab activates NK cells and MyD88-dependent Toll-like receptor (TLR) signaling which stimulates the release of type I IFNs and then primes adaptive IFN*γ*-producing CD8+ T cells. In addition, trastuzumab, through its Fc region, increases HER2 uptake by dendritic cells, facilitating cross-presentation of HER2 peptides and activation of antigen-specific T cells [28]. In support of this model, intratumoral administration of CpG and poly I:C, agonists of TLR9 and TLR3 on NK cells, synergizes with trastuzumab in the treatment of HER2-positive tumors to creating local inflammatory conditions that are necessary for lymphocytic infiltration and trastuzumab activity [29]. These compounds augment NK cell-mediated ADCC and IFN responses, triggering acquired and long-lasting antitumor immunity that is centered on CD8+ T cells and IFN*γ* [29]. The same benefit has been obtained using polysaccharide-K, a potent agonist of TLR2 that activates NK cells and potentiates trastuzumab-mediated ADCC [30].

Mortenson et al. demonstrated that also CD4+ T cell participates in anti-HER2 therapy: CD4 depleting antibodies reduce antitumor activity of anti-HER2 MAbs that exert their effect also through a CD4-dependent antitumor-specific response (Figure 1). Upon treatment, CD4+ T cells are recruited in the tumors, enhance and maintain CD8+ T cell activation, and induce MHC-II expression on tumor cells through the release of IFN*γ*, leading to their recognition and death [27]. CD4+ T-helper lymphocytes, which secrete type I cytokines, such as IFN*γ* and tumor necrosis factor *α* (TNF*α*), contribute to the induction of a cytotoxic antitumor response that cooperates with trastuzumab to upregulate MHC-I on HER2-positive BC cells, facilitating their recognition and lysis by CD8+ lymphocytes [31]. Accordingly, patients who benefit from trastuzumab treatment in neoadjuvant settings have more extensive anti-HER2 CD4+ T cell responses by IFN*γ* enzyme-linked immunosorbent spot analysis (ELISPOT) than non-pCR patients after treatment [32]. Moreover, trastuzumab-induced IL21 expression in CD4+ T lymphocytes drives CD8+ T cell antitumor responses against HER2-positive tumors [33]. Signaling downstream of IL21R is important in CD8+ T cell activity, and recombinant IL21 improves the therapeutic efficacy of anti-HER2 MAb. A recent study has suggested that intratumoral delivery of IL21, in combination with anti-HER2 mAb therapy, enhances the therapeutic effects of trastuzumab, skewing tumor-associated macrophages away from a M2 phenotype to a tumor-inhibiting M1 phenotype [34]. Per the IL21-IL21R axis, greater IL21R expression in tumor tissues from patients in the FinHER trial was associated with benefit of trastuzumab with regard to distant relapse [33].

Although trastuzumab significantly increases anti-HER2 humoral responses (against the extracellular and intracellular domains) primarily in metastatic patients with objective responses [35, 36], preclinical data suggest that secreted antibodies do not contribute mechanistically to clinical outcomes, because B cell depletion does not affect trastuzumab activity in preclinical models [27]. These data suggest that the production of anti-HER2 antibodies, mainly in patients who benefit from treatment, merely reflects and confirms activation of the adaptive immune response on trastuzumab treatment.

The monoclonal antibody pertuzumab (Perjeta, Hoffmann-La Roche, Basel, Switzerland) binds to the extracellular domain of HER2 and prevents the ligand-mediated dimerization of HER2 with HER1 and HER3 by steric hindrance [37] (Figure 1). In particular, this agent is more effective than trastuzumab in disrupting HER2-HER3 complex formation, and its efficacy is maintained in cells that express low levels of HER2 [38]. In contrast to trastuzumab, pertuzumab does not prevent HER2 ECD shedding and is unable to inhibit dimerization in a ligand-independent manner [37]. Sims and colleagues reported that pertuzumab mediates ADCC, observing an increase in immune-related genes on treatment in ovarian cancer [39], and HER2-overexpressing Calu-3 and KPL-4 cells are killed by pertuzumab-mediated ADCC *in vitro* [40].

Based on its synergistic effects when combined with trastuzumab versus alone as a monotherapy [40, 41], most preclinical studies on pertuzumab have focused on cotreatment with trastuzumab [41, 42]. The combination of trastuzumab and pertuzumab inhibits cell proliferation and survival and induces apoptosis to a greater degree than either individual agent [41]. Moreover, this combination increases the disruption of HER2 dimers and impedes signaling in the Akt cell survival pathway [41].

ADCC is one of the most important mechanisms of action of trastuzumab and pertuzumab, as evidenced by the rise in NK cells that infiltrate and penetrate deeper into the tumor burden of mice that harbor trastuzumab-resistant JIMT-1 cells and have been treated with trastuzumab and pertuzumab compared with mice that have been given each agent individually [42]. Based on data on trastuzumab, it is also likely that adaptive immunity is crucial for the synergism between trastuzumab and pertuzumab. Moreover, the combination of these antibodies activates complement-mediated cytotoxicity (CDC), which is poorly engaged by trastuzumab and pertuzumab alone [43]. Only with the combination treatment cells are likely to have a sufficient number of cell-bound antibodies nearby to bind and activate C1q, which is required to initiate the complement cascade, as suggested by the extensive CDC that is observed by targeting of multiple HER2 epitopes with several monoclonal antibodies [44].

### 2.2. Tyrosine Kinase Inhibitors

Many tyrosine kinase inhibitors (TKIs) have activity in trastuzumab-resistant BC and can be used as alternatives to block HER2 signaling. Among such emerging HER TKIs [45], lapatinib and neratinib have been approved by the FDA for HER2-positive BC. Lapatinib (GW572016, Tyverb/Tykerb; Novartis, Switzerland) reversibly inhibits the intracellular tyrosine kinase domains of EGFR and HER2, inducing cell cycle arrest and apoptosis in BC cell lines through the cleavage of PARP and the activation of caspase 3 [46]. Lapatinib also blocks BC cell proliferation robustly in trastuzumab-resistant cell line concentration dependently. In cells with HER2 gene amplification, sensitivity to lapatinib is associated with higher overexpression of HER2 and EGFR [47]; thus, sensitivity to lapatinib has been used to identify BC cell lines that are dependent on the HER2 oncogene for growth (HER2 addicted) [48].

Lapatinib downregulates phospho-HER2 (p-HER2), p-EGFR, and p-ERK and promotes mutant p53 degradation *in vitro* and *in vivo* by inhibiting HSF1 (heat shock transcription factor 1) and its target, HSP90 [49]. In addition, lapatinib heightens the sensitivity of cells to radiation, delaying DNA repair mechanisms, as reflected by increases in radiation-induced *γ*H2AX foci [50]. The antitumor activity of lapatinib has been demonstrated in xenograft models, and its combination with trastuzumab has additive and synergistic inhibitory effects on growth [47]. In conjunction with tamoxifen, lapatinib induces more extensive cell cycle arrest through rises in p27 and downregulation of estrogen receptor (ER) transcriptional activity [51].

By blocking the tyrosine kinase domain of HER2, lapatinib elicits the accumulation of HER2 on the cell membrane [52] in the BC cell lines BT474 and SKBr3, increasing trastuzumab-dependent cytotoxicity in combination with trastuzumab [53] (Figure 1). Accordingly, a second treatment round of trastuzumab on lapatinib administration reduced the tumor burden in a case study of metastatic HER2-positive BC that was resistant to anti-HER2 antibody [53]. Thus, the lapatinib-induced upregulation of HER2 on the cell surface has the potential to convert refractory tumors into trastuzumab-sensitive tumors. In addition to increasing trastuzumab-mediated ADCC, an immune-related mechanism of action has been suggested for lapatinib. In MMTV-neu animals, lapatinib promotes tumor infiltration of IFN*γ*-secreting CD4+ and CD8+ T cells in a Stat1-dependent manner [54]. In contrast, lapatinib is less effective in Stat1-deficient mice, likely due to the impaired proliferation of IFN*γ*-secreting CD8+ cells.

Neratinib (HKI-272, Puma Biotechnology Inc., Los Angeles, CA, USA) is an oral pan-HER inhibitor that bonds covalently with a conserved cysteine residue (Cys-773) in the kinase domain of HER, leading to its irreversible inhibition and thus blocking the pathways that lie downstream of EGFR, HER2, and HER4 (Figure 1). Its specificity for Cys-773 renders neratinib highly selective for HER family members [55]. By binding its target, neratinib prevents the activation of the 4 HER receptors in HER2-positive BC and inhibits downstream pathways, causing G0/G1 cell cycle arrest and thus inhibiting proliferation in tumor cells *in vitro* and in cells with innate and acquired trastuzumab resistance [56, 57]. Like lapatinib, the antitumor efficacy of neratinib correlates with HER2 expression and activation, and neratinib is inactive in tumor cells that express low levels of HER2 and EGFR [56, 57]. Neratinib downregulates HER2 levels on the cell surface [57], but the influence of neratinib on trastuzumab-mediated cell cytotoxicity remains under investigation [58].

## 3. Efficacy of HER2-Targeting Agents in Neoadjuvant and Adjuvant Settings

### 3.1. Trastuzumab

Trastuzumab was approved for metastatic HER2-positive BCs in 1998 by the FDA after a phase III trial demonstrated that its addition to standard chemotherapy extended the time to progression from 4.6 to 7.4 months and reduced the relative risk of death by 20% [59].

Neoadjuvant treatment with trastuzumab and chemotherapy was introduced into clinical practice, based on results of 3 phase III trials that recorded higher pathologic complete response (pCR) rates in the trastuzumab arm (Table 1), compared with the same chemotherapy alone, and improved disease-free survival (DFS) [22, 60, 61]. A meta-analysis of 5 randomized trials, comprising 515 patients, concluded that the addition of trastuzumab to chemotherapy for HER2-positive BC in the neoadjuvant setting improves the likelihood of achieving a higher pCR rate (odds ratio (OR): 1.85, 95% CI: 1.39–2.46; *p* value < 0.001) with no additional toxicity [62]. Moreover, an exploratory pooled analysis of 8 German neoadjuvant studies—randomized and nonrandomized—of 614 patients showed a 3.2-fold improvement in pCR (OR: 3.2, 95% CI: 2.19–4.67; *p* value < 0.001) in HER2-positive patients who received trastuzumab versus those who did not [63]. Also, in the actual clinical treatment of HER2-positive BC, the pCR rate (46.8%) to trastuzumab with various chemotherapy regimens is similar to that in the clinical trials (approximately 40%) [64].

In the adjuvant setting, based on the results of 4 large trials (HERA [65], FinHER [66], NCCTG N9831, and the NSABP B-31 trials [67], Table 1), trastuzumab is recommended as monotherapy for 1 year after completion of chemotherapy, in combination with taxanes on completion of doxorubicin plus cyclophosphamide and concurrently with carboplatin and docetaxel [68]. A meta-analysis of 4 randomized clinical trials and BCIRG 006 [69] (*n* = 13493 women) showed that survival in trastuzumab-treated patients was superior in terms of DFS (risk ratio (RR): 0.62; 95% CI: 0.56–0.68) and mortality (RR: 0.66; 95% CI: 0.57–0.77) [70].

In analyses of “real-world” treatment, patients who were given trastuzumab for early-stage HER2-positive BC had 5-year DFS and OS rates that were comparable with those in randomized trials. Of 476 patients in the Netherlands, those who were treated with trastuzumab had a superior DFS (adjusted HR = 0.63, 95% CI = 0.37–1.06) than subjects who underwent chemotherapy alone [71]. A retrospective Italian study, GHEA, which analyzed 1002 patients who were treated per the HERA protocol, recorded 107 BC relapses (overall frequency, 10.67%), with a 3-year DFS of 87% [72], similar to what was observed in the HERA trial (4-year DFS: 78.6%). A similar study in 313 patients in Slovenia reported an 81% 4-year DFS and a 92% OS with trastuzumab plus chemotherapy [73].

### 3.2. Pertuzumab

Pertuzumab was approved by the FDA in 2012 for use in combination with trastuzumab and docetaxel for patients with HER2-positive MBC who have not received prior anti-HER2 therapy or chemotherapy for metastatic disease, based on a multicenter, randomized, double-blind, placebo-controlled trial (CLEOPATRA) in which its addition to trastuzumab and docetaxel improved progression-free survival (HR = 0.62; 95% CI: 0.51–0.75; *p* < 0.0001) [74]. In 2013, based on the open-label phase II NeoSphere trial and TRYPHAENA phase II study, accelerated FDA approval for pertuzumab in combination with trastuzumab and docetaxel for early-stage BC was obtained [75]. The NeoSphere trial demonstrated that dual blockade with trastuzumab, pertuzumab, and docetaxel increased pCR rates compared with trastuzumab and docetaxel (45.8% versus 29%, resp.) [76], whereas the TRYPHAENA study reported pCR rates of 57% to 66% with various chemotherapeutic regimens in conjunction with trastuzumab and pertuzumab [77] (Table 1).

Positive results were recently published for the phase III APHINITY trial, comparing the activity of adjuvant pertuzumab, trastuzumab, and chemotherapy versus trastuzumab and chemotherapy. The study met its primary endpoint and showed that the dual blockade approach effected a statistically significant reduction in the risk of recurrence of invasive disease or death compared with trastuzumab and chemotherapy alone (3-year DFS HR = 0.81; 95% CI: 0.66–1.00; *p* = 0.045), despite the 3-year DFS rising modestly from 93.2% to 94.1% [78].

### 3.3. Lapatinib

Lapatinib received approval by the FDA in 2007 for metastatic BC (MBC), based on a phase III study that compared lapatinib/capecitabine with capecitabine alone in patients with MBC who progressed after chemotherapy/trastuzumab, in which TTP improved from 4.4 to 8.4 months [79]. Lapatinib is now used in combination with the chemotherapeutic agent capecitabine, primarily as a second line treatment. Based on data that lapatinib with chemotherapy is less active than trastuzumab plus chemotherapy for HER2-positive MBC (reviewed in [80]), the study of the efficacy of lapatinib in early settings has focused primarily on its combination with trastuzumab.

Dual blockade of HER2 with trastuzumab and lapatinib has been examined in the neoadjuvant treatment setting in 4 randomized studies (Table 1), comparing the activity of trastuzumab, lapatinib, or both with paclitaxel: the phase III NeoALTTO [81], the phase II CHER-LOB [82], the phase III NSABP B-41 [83], and the phase III CALGB 40601 [84]. Dual blockade was superior to trastuzumab alone with regard to pCR in all studies but significantly in only NeoALTTO and CHER-LOB. A meta-analysis that included data from these trials concluded that the addition of lapatinib to trastuzumab improves the probability of achieving a pCR compared with trastuzumab alone (RR: 1.39, 95% CI 1.20–1.63; *p* < 0.001) (779 patients) [85]. Although these studies showed no significant difference between the lapatinib and trastuzumab arms in terms of pCR, a meta-analysis of 1494 patients demonstrated that the probability of achieving a pCR was higher for the trastuzumab plus chemotherapy versus lapatinib plus chemotherapy arm (RR: 1.25, 95% CI: 1.08–1.43; *p* = 0.003) [85].

Dual blockade in the adjuvant setting with trastuzumab and lapatinib in combination with taxanes was tested in 8381 women in the phase III ALTTO trial, but the 16% improvement in DFS (HR = 0.84, 95% CI = 0.70–1.12, *p* = 0.0480) with concomitant dual blockade compared with trastuzumab alone was not statistically significant (*p* ≤ 0.025 was required for statistical significance in the test for superiority of the lapatinib plus trastuzumab versus trastuzumab arm) [86].

### 3.4. Neratinib

Neratinib has been investigated in all settings (reviewed in [87]). In the metastatic setting, the efficacy of neratinib appears to be similar to that of trastuzumab when combined with taxanes, suggesting that it is superior to its parent compound, lapatinib. The ongoing randomized phase III NALA trial is comparing the combination of capecitabine plus neratinib or lapatinib, and the results of which might alter the clinical management of MBC. Also, in the neoadjuvant setting, neratinib has demonstrated promising results in a phase II study (I-SPY 2 [88]), effecting a 39% pCR versus 23% with trastuzumab.

In the adjuvant setting, neratinib has recently been approved by the FDA for extended treatment of early-stage HER2-positive BC [89], based on the results of the phase III ExteNEt trial, which reported a small but significant improvement in 2-year DFS in women who received it after adjuvant trastuzumab versus placebo (93.9% versus 91.6%, HR = 0.67, 95% CI: 0.50–0.91, *p* = 0.0091) [90]. Notably, contrary to what has been observed for pertuzumab and lapatinib, hormone receptor- (HR-) positive patients derived a greater benefit from neratinib (HR = 0.51, 95% CI: 0.33–0.77, *p* = 0.0013) than HR-negative patients (HR = 0.93, 95% CI: 0.60–1.43, *p* = 0.74).

All current data (reviewed in [87]) suggest that neratinib is a promising drug for the treatment of BC patients with HER2-positive tumors and merits further development in the metastatic and adjuvant/neoadjuvant settings.

## 4. Biomarkers of Response to HER2-Targeting Agents

Given the availability of effective agents, biomarkers that differentiate patients who actually need new adjuvant therapies must be identified. Several efforts have been made in the last decade to discover biomarkers that predict who might benefit from trastuzumab, but most have failed to be consistently validated in tumor samples from randomized clinical trials (reviewed in [91]).

New high-throughput genomic technologies have increased the rate of discovery of potential markers with prognostic or predictive value. These technologies demonstrated the intrinsic molecular heterogeneity in clinically HER2-positive BCs. The PAM50 classifier identified all of the intrinsic subtypes in HER2-amplified BCs, 50% of which are classified HER2-enriched (HER2-E) [92]. These tumors experience the most extensive activation of the HER2/EGFR signaling pathway [93], suggesting that they depend on the HER2 receptor and benefit the most from trastuzumab. The application of PAM50 predictor of tumors to the major neoadjuvant clinical trials of anti-HER2 agents (NOAH, CALGB 40601, NeoALTTO, and CHER-LOB) found that patients with HER2-E tumors benefited substantially from a trastuzumab-based treatment, achieving a significantly higher pCR rate than those with other tumors [84, 94–96] (Table 1). Notably, in the NeoALTTO trial, PAM50 had a significant effect on pCR across arms [95], similar to that observed in the CALGB 40601 trial [84], supporting its predictive value for both trastuzumab and lapatinib. In the adjuvant phase III NSABP B-31 trial, PAM50 failed to identify subgroups that benefited differentially from trastuzumab [97], whereas in the NCCTG-N9831 trial, patients with HER2-E or luminal tumors benefited from the addition of trastuzumab to chemotherapy, unlike those with basal-like tumors [98], suggesting the need to further evaluate this predictor in the adjuvant setting.

A retrospective analysis of the NSABP B-31 study indicated that *ERBB2* and *ESR1* mRNA levels influence the degree of benefit that is received from adjuvant trastuzumab [99]. Similarly, the trastuzumab risk (TRAR) prediction model, based on expression levels of 41 genes that are related to *ERBB2* and *ESR1*, is predictive of early relapse in adjuvant setting [100]. In the neoadjuvant setting, *ESR1* and *ERBB2* levels, as determined by mRNAseq, when considered as continuous variables, were individually linked to pCR, and their incorporation into an exploratory multivariate model removed intrinsic subtype, HER2 amplicon signature, and clinical assays for ER or HER2 from the model in the CALGB 40601 trial [84]. The levels of *ERBB2* and *ESR1* were confirmed in the NeoALTTO trial across arms [95] as the most important determinants of pCR compared with standard tests, in the GeparQuattro [101] and in the TRYPHAENA trials [77] (Table 1). Additional evidence is needed before *ESR1* and *ERBB2* RNA can be implemented in the clinical setting, but their predictive ability in HER2-positive BC supports the superiority of evaluating their mRNA levels over standard IHC tests and suggests that they better mirror activity of HER2 and tumor-addiction to its downstream signals, as PAM50 did.

Llombart-Cussac et al. have attempted to modify therapy according to the intrinsic features of the tumor, determining the value of intrinsic molecular subtypes in predicting pCR in patients with HER2-positive BC following neoadjuvant dual blockade with trastuzumab and lapatinib in the absence of chemotherapy in the PAMELA trial [102]. Patients who achieved a pCR had HER2-E tumors in 89% of cases confirming the higher sensitivity of HER2-E tumors to anti-HER2 agents and supporting the possibility of deescalating treatment by removing chemotherapy, at least in a subgroup of HER2-E patients (41% of HER2-E tumors attained a pCR). However, that only approximately half of HER2-E tumors benefit from anti-HER2 agents, with or without chemotherapy, indicates that intrinsic features—even if they are relevant—are insufficient for predicting whether one will benefit from anti-HER2 treatment.

The relevance of the immune system in trastuzumab activity has prompted several groups to examine the use of immune status to identify patients who are likely to benefit from trastuzumab (Table 1). Perez et al. [103] developed a genomic signature that predicts who will benefit from trastuzumab in samples of the NCCTG N9831 trial, consisting of 14 immune-related genes and classifying tumors as immune response-enriched (IRE) and nonimmune response-enriched (NIRE). Only patients with IRE tumors that were enriched in genes that are related to T and B cell responses, chemokine signaling, and inflammation had an increased DFS when treated with trastuzumab. Application of this signature in the NeoALTTO trial associated positively with a pCR, like other interferon-related signatures that are highly correlated with IRE expression [95].

The expression of immune genes and metagenes has been also correlated with pCR in the NeoSphere and NOAH trials, in which, for example, the STAT1 and MHC-I metagenes were linked to higher and lower pCRs, respectively [104]. Accordingly, high infiltration of tumor-infiltrating lymphocytes (TILs) was consistently associated with a higher pCR in the NeoALTTO [105], CHER-LOB [96], and NeoSphere trials [104]. Also, in a combined analysis of the GeparQuattro [61] and GeparQuinto [106] trials, HER2-positive lymphocyte-predominant BC (LPBC) cases, with more than 50% TILs, had significantly higher pCR rates compared with non-LPBC types [107].

In the adjuvant setting, the association between immune-related biomarkers and DFS is more controversial. Loi et al. [108] reported an association between TILs and benefit from trastuzumab treatment with regard to DFS in the FinHER trial [108], whereas in the NCCTG N9831 trial, pathological evaluation of TILs was not predictive of a benefit of trastuzumab but was associated with a benefit from chemotherapy [109]. Also, the IRE signature, although it was developed in the adjuvant setting, failed to predict a benefit from trastuzumab in the NSABP B-31 trial [110], supporting the definitive exploration of these biomarkers in large adjuvant trials (ALTTO and APHINITY).

Analysis of immune-related biomarkers in the NeoALTTO trial showed that TIL levels were associated with higher pCR rates, independent of treatment arm [105], whereas the positive effect of the immune signatures on pCR was specific to the dual blockade arm (trastuzumab, lapatinib, and taxanes), despite the trend being similar in all arms [95]. In contrast, in the NeoSphere trial, the predictive abilities of immune genes differed between treatment arms [104]—higher expression of all immune metagenes correlated with a lower probability of pCR in the dual blockade arm (trastuzumab, pertuzumab plus taxanes), whereas high levels of various immune markers were associated with a greater likelihood of pCR in the other 3 arms. Accordingly, in this trial, the pCR rate in the dual blockade versus other arms was higher in the group with low and intermediate TILs but not in the LPBC groups, suggesting that patients with low immune infiltration benefit most from this treatment. Based on these data, immune genes are potential biomarkers that can be used to identify patients who are likely to benefit from trastuzumab (high infiltrate) and those who might benefit from the addition of pertuzumab (low infiltrate).

TIL levels at baseline were also associated with better outcomes, independent of treatment arm [105, 107], further supporting the possibility of treating tumors with high TIL levels solely with the current standard of trastuzumab and chemotherapy—that is, without the addition of dual blockade agents—once the ideal cutoff of TIL levels is identified for the clinical management. Notably, patients who did not achieve pCR and had low basal levels of TILs had the poorest survival in the NeoALTTO and GeparQuattro/Quinto trials [105, 107], suggesting that additional therapeutic strategies—for example, immune-enhancing approaches—are needed for this subgroup of patients. Conversely, those who reached a pCR and had high TIL levels had an excellent prognosis, supporting the addition of TIL level to pCR as a prognostic marker after neoadjuvant therapy with anti-HER2 agents. These data other than indicating an association between basal infiltration of tumor by TIL and the benefit to HER2-targeted agents support also the prognostic power of this biomarker in HER2-positive tumors independently from treatment.

The predictive power of immune-related features in tumor biopsies before neoadjuvant treatment with regard to pCR (Table 1) and DFS suggests that trastuzumab can induce an antitumor vaccinal effect in responsive patients, as it was observed in preclinical models. On the contrary, the inability of these biomarkers to predict DFS in the adjuvant setting (Table 1), when the treatment acts against circulating cells and/or micrometastasis, indicates that for an optimal vaccinal-like effect, the tumor must be around during treatment to achieve permanent tumor eradication. Altogether these findings render immune-related markers at the forefront of biomarkers that warrant application in clinical practice and for future drug development in HER2-positive BC. In addition, based on their discriminatory predictive power, according to the drug that is used, they could guide patients toward the most appropriate treatment option. The immune response is a complex process that involves many components with antitumor activity and protumor effects, due to the immune-escape state that is established. Thus, it is likely that more refined evaluations of tumor-associated and circulating immune responses will result in even better immune biomarkers.

Moreover, based on the immune modulating ability of trastuzumab, the evaluation of TIL levels/immune markers in the residual disease after neoadjuvant therapy and their alteration from the baseline could add relevant information on the pharmacodynamic modulation of the immune microenvironment by trastuzumab. Another emerging approach for identifying new predictive biomarkers exploits the brief exposure paradigm. In 2 phase II trials (03-311 and 211B) [111], single-dose trastuzumab increased immune-related gene expression, primarily in HER2-E tumors, and the expression of CD4+ T cell-related metagene on exposure to trastuzumab was predictive of the response to neoadjuvant trastuzumab and chemotherapy, supporting the early evaluation of the therapeutic response to trastuzumab and providing an opportunity for triage to the dual blockade therapy for unresponsive patients.

## 5. Future Perspectives

Although much work remains in the effort to refine and optimize biomarkers predictive of trastuzumab/HER2 double-blockade benefits and their application in patients, the reported robustness of immune infiltration/TIL evaluation (Table 1) and the known clinical benefits of antibody therapy justify such efforts. Future clinical studies in HER2+ subtype should consider TILs/immune genes other than tumor intrinsic characteristics as a stratification factor and investigate whether therapies that can augment immunity could potentially further improve survival.

The implementation of immune markers in everyday clinical practice requires robust assessment of their clinical utility and of the test analytical and clinical validity, other than the understanding of their added predictive value, if any, on tumor intrinsic characteristics (e.g., PAM50) and the identification of the best performing immune-related marker. Indeed, TIL evaluation on H&E slides is an easy method to provide raw information on the complexity of the tumor immune microenvironment, but it does not give information on the composition and functional status of the immune infiltrate that can be obtained using immune gene signatures, as evaluated by mRNA profiling. Several efforts have been made to standardize TIL assessment, and guidelines have been published and continuously improved [112, 113]. The association between TIL infiltration in HER2-positive tumors and patient good prognosis supports clinical utility of TIL quantification at least to identify patients eligible for treatment deescalation to taxane and trastuzumab alone [114].

In an attempt to understand the clinical utility of immune markers in predicting response to trastuzumab in CHERLOB study, TILs failed to provide an independent prediction of pCR beyond PAM50 and were outperformed by immune-related gene signatures [96]. These data suggest that intrinsic molecular subtypes and immune gene signatures that mirror T cell infiltration/activation (T cell/immune 2) and antigen processing/presentation (immune 3) provide distinct biological information independently affecting sensitivity to anti-HER2 therapy, supporting the integration of such predictive biomarkers.

Several key questions remain regarding the immune-biology of HER2-positive tumors that could help in developing new strategies to modulate immune response and improve anti-HER2 therapy efficacy; what drives immune infiltration in tumors is an ongoing area of research. It has been hypothesized that immune infiltrate is dictated by high mutational burden corresponding to a greater amount of neoantigens. Although cancer neoantigens are required for mounting an anticancer immune response, recent evidence showed that mutational burden does not correlate with the presence or absence of CD8+ T cells in the tumor microenvironment of melanoma and with T cell signature in any cancer type [115]. Accordingly, no significant correlation between the amount of neoantigens arising from tumor somatic mutation and TIL count or survival upon trastuzumab treatment was found in HER2-positive BC of the FinHER trial [116], indicating that spontaneous immune infiltration in tumor is unlikely to be completely dependent on neoantigen count. Infiltration of tumors by T cells could instead be associated with oncogene activation, as demonstrated for other oncogenes such as RET/PTC in thyroid cancer [117], RAF in melanoma, and MYC in pancreatic tumors [118]. In support of this hypothesis, we demonstrated that TRAR-low patients, who are sensitive to trastuzumab treatment, are those with both tumor dependence on HER2 signaling by PAM50 classification and enrichment in immune genes and CD8+ T cells, supporting a direct connection between these two features [100]. We found that HER2/ER activity shaped the tumor immune microenvironment regulating chemokine expression and PD-1 ligands [119], suggesting that tumor cells could directly mold their microenvironment. This speculation is also supported by the fact that immune microenvironment of the primary tumor is predictive of trastuzumab benefit both in neoadjuvant and in adjuvant setting, when the primary tumor has been surgically removed and therapy is directed against micrometastatic tumor foci.

Independently from TIL recruitment mechanisms, another important issue in the prospect of augmenting trastuzumab activity by strategies of immune modulation regards the activation status of such infiltrating immune cells. It is possible that the presence of TILs in the tumor burden mirrors an exhausted immune response, the presence of intratumoral immune suppression or a near-equilibrium immune state with immune surveillance able to only partially control the tumor growth of immunogenic subclones slowing the genomic diversification of the cancer [120]. In support of the last hypothesis, it has been observed that cancers with no immune infiltration have greater clonal heterogeneity, likely suggesting an immune escape [120].

Efficacy of drugs in those patients with immune-enriched tumors suggests that chemotherapy and HER2-targeting agents may relieve the preexisting immune suppression and/or tilt the balance in favor of immune surveillance [120]. In this context, a number of chemotherapies including anthracyclines, gemcitabine, oxaliplatin, and cyclophosphamide have been shown to increase antigen presentation promoting DC maturation and priming of adoptive immune response [121, 122]. These data underlie the importance of immunogenic cell death (ICD) for immune system activation and antitumor response and of the selection of chemotherapy agents to be used in combination with anti-HER2 therapy in order to reach the best immune activation and thus the best achievable response. It is noteworthy that the double-blockade treatment (pertuzumab or lapatinib in combination with trastuzumab) induced pCR rates similar to trastuzumab plus anthracycline (Table 1), an effect that probably relies in the ability of the double blockade to boost the immune response (i.e., increased ADCC and CDC) [42, 43, 53, 54]. These data support the double-blockade treatment to limit the anthracyclines-associated cardiovascular toxicity at least in women at high risk of cardiac toxicity.

To activate antitumor immunity, recent preclinical data suggested that one strategy could be the blocking of PD-1/PD-L1 interaction, since it synergizes with anti-HER2 therapy [25]. Accordingly, a large number of clinical trials are now under way to determine the clinical role of immunotherapies and their combinations with anti-HER2 therapies in BC [123]. However, breast tumors with a low number of TILs are less likely to achieve maximal clinical benefit from anti-HER2 agents combined with T cell checkpoint inhibitors and, although many efforts have been done so far to study the possibility to activate the local innate IFN response [29] or to contrast immune evasion [124], different strategies to stimulate the tumor immune milieu still need to be investigated.

## Figures and Tables

**Table 1:** Evaluation of HER2-addiction and immune biomarkers in randomized trials investigating anti-HER2 targeted therapies.

Drug	Setting	Trial	Treatment	pCR, % or DFS, HR (95% CI)	Addiction^†^	Immune^Δ^
Trastuzumab (H)	Neoadjuvant	Buzdar et al. [22]	FEC FECH	26 65^∗^	—	—
	Neoadjuvant	NOAH [60]	AP > P > CMF APH > PH > CMFH	22 43^∗^	Yes [94]	Yes [104]
	Neoadjuvant	GeparQuattro [61]	EC > D EC > DH	16 32^∗^	Yes [101]	Yes [107]
	Adjuvant	BCIRG 006 [69]	AC > D AC > DH > H	1 0.64 (0.53–0.78)^∗^	—	—
	Adjuvant	FinHER [66]	D(V) > FEC D(V)H > FEC	1 0.42 (0.21–0.83)^∗^	—	Yes [108]
	Adjuvant	HERA [65]	Ch Ch > H	1 0.76 (0.67–0.86)^∗^	—	—
	Adjuvant	NSABP B-31 [67]	AC > P AC > PH	1 0.52 (0.45–0.6)^∗^	No [97] Yes [99]	No [110]
	Adjuvant	NCCTG N9831 [67]	AC > P AC > PH	1 0.52 (0.45–0.6)^∗^	—	Yes [103] No [109]

Trastuzumab (H) and/or lapatinib (L)	Neoadjuvant	CHER-LOB [82]	PH > FECH PL > FECL PHL > FECHL	25 26 47^∗^	Yes [96]	Yes [96]
	Neoadjuvant	CALGB 40601 [84]	PH PL PHL	46 32 56^∗^	Yes [84]	—
	Neoadjuvant	GeparQuinto [106]	ECH > DH ECL > DL	30 23^∗^	—	Yes [107]
	Neoadjuvant	NeoALTTO [81]	PH PL PHL	29 25 51^∗^	Yes [95]	Yes [105]
	Neoadjuvant	NSABP B-41 [83]	AC > PH AC > PL AC > PHL	52 53 62	—	—
	Adjuvant	ALTTO [86]	PH PHL	1 0.84 (0.7–1.12)	—	—

Trastuzumab (H) and/or pertuzumab (Pz)	Neoadjuvant	NeoSphere [76]	DH DPz HPz DHPz	29 24 17 46^∗^	Yes [13]	Yes [104]
	Neoadjuvant	TRYPHAENA [77]	FEC > DHPz FECHPz > DHPz CDHPz	57 62 66	Yes [12]	—
	Adjuvant	APHINITY [78]	C(F)E > TH C(F)E > THPz	1 0.81 (0.66–1.00)^∗^	—	—

^∗^The comparison is statistically significant; ^†^HER2-addiction as evaluated by PAM50 or *ERBB2*/*ESR1* gene expression; ^Δ^immune-related features as evaluated by TIL count or immune metagene. In these columns, it is indicated whether the biomarker is significantly associated (yes) or not (no) with outcome; A: adriamycin; C: cyclophosphamide; Ch: chemotherapy; D: docetaxel; DFS: disease-free survival; E: epirubicin; F: fluorouracil; H: trastuzumab; L: lapatinib; M: metotrexate; P: paclitaxel; pCR: pathological complete response; Pz: pertuzumab; T: taxanes; V: vinorelbine.
